# New onset or relapsing neuromyelitis optica temporally associated with SARS-CoV-2 infection and COVID-19 vaccination: a systematic review

**DOI:** 10.3389/fneur.2023.1099758

**Published:** 2023-06-22

**Authors:** Tamar Harel, Emily F. Gorman, Mitchell T. Wallin

**Affiliations:** ^1^Department of Veterans Affairs Multiple Sclerosis Center of Excellence (VA MSCoE), Baltimore VA Medical Center, Baltimore, MD, United States; ^2^Department of Neurology, University of Maryland Medical Center, Baltimore, MD, United States; ^3^Health Sciences and Human Services Library, University of Maryland, Baltimore, MD, United States

**Keywords:** neuromyelitis optica, COVID-19, severe acute respiratory syndrome coronavirus 2, COVID-19 vaccine, outcomes

## Abstract

**Background:**

Neuromyelitis optica spectrum disorder (NMOSD) is a rare chronic neuroinflammatory autoimmune condition. Since the onset of the COVID-19 pandemic, there have been reports of NMOSD clinical manifestations following both SARS-CoV-2 infections and COVID-19 vaccinations.

**Objective:**

This study aims to systematically review the published literature of NMOSD clinical manifestations associated with SARS-CoV-2 infections and COVID-19 vaccinations.

**Methods:**

A Boolean search of the medical literature was conducted between December 1, 2019 to September 1, 2022, utilizing Medline, Cochrane Library, Embase, Trip Database, Clinicaltrials.gov, Scopus, and Web of Science databases. Articles were collated and managed on Covidence^®^ software. The authors independently appraised the articles for meeting study criteria and followed PRISMA guidelines. The literature search included all case reports and case series that met study criteria and involved NMOSD following either the SARS-CoV-2 infection or the COVID-19 vaccination.

**Results:**

A total of 702 articles were imported for screening. After removing 352 duplicates and 313 articles based on exclusion criteria, 34 articles were analyzed. A total of 41 cases were selected, including 15 patients that developed new onset NMOSD following a SARS-CoV-2 infection, 21 patients that developed *de novo* NMOSD following COVID-19 vaccination, 3 patients with known NMOSD that experienced a relapse following vaccination, and 2 patients with presumed Multiple Sclerosis (MS) that was unmasked as NMOSD post-vaccination. There was a female preponderance of 76% among all NMOSD cases. The median time interval between the initial SARS-CoV-2 infection symptoms and NMOSD symptom onset was 14  days (range 3–120  days) and the median interval between COVID-19 vaccination and onset of NMO symptoms was 10  days (range 1 to 97  days). Transverse myelitis was the most common neurological manifestation in all patient groups (27/41). Management encompassed acute treatments such as high dose intravenous methylprednisolone, plasmapheresis, and intravenous immunoglobulin (IVIG) and maintenance immunotherapies. The majority of patients experienced a favorable outcome with complete or partial recovery, but 3 patients died.

**Conclusion:**

This systematic review suggests that there is an association between NMOSD and SARS-CoV-2 infections and COVID-19 vaccinations. This association requires further study using quantitative epidemiological assessments in a large population to better quantify the risk.

## Introduction

Novel coronavirus disease (COVID-19), a respiratory disease caused by severe acute respiratory syndrome coronavirus 2 (SARS-CoV-2), was first detected in Wuhan, China in December 2019, and by March 2021 the World Health Organization (WHO) declared a worldwide pandemic (1). As of November 2, 2022, globally there have been over 628 million confirmed cases of COVID-19, including over 6.57 million deaths, and over 12.85 billion doses of the vaccine have been administered (2). Despite the rapid development and distribution of vaccinations, COVID-19 remains a prevalent and serious public health condition today.

SARS-CoV-2 has the ability to dysregulate the host immune system, producing various autoantibodies (3–5). This can induce a cascade of immune-mediated central nervous system (CNS) damage from either direct inoculation of the CNS or a systemic autoimmune response toward the virus (3–6). It has been shown that SARS-CoV2 can traverse the blood brain barrier and provoke CNS demyelination (3). Given this background, it is not surprising that a variety of case reports have linked the SARS-CoV-2 infection with an array of CNS autoimmune demyelinating disorders such as transverse myelitis (TM), acute demyelinating encephalomyelitis (ADEM), multiple sclerosis (MS), and neuromyelitis optica spectrum disorder (NMOSD) (6–8).

NMOSD is a chronic, relapsing, autoantibody-mediated astrocytopathy channelopathy that presents as severe CNS demyelination attacks commonly involving TM, optic neuritis (ON), area postrema syndrome (APS), and acute brainstem syndrome (BS) (9). The underlying pathogenic mechanism that leads to NMOSD is unclear, but mounting evidence suggests that there is an intricate interplay between environmental factors, such as vaccines and viral infection, and genetic susceptibility that leads to CNS inflammation (10–12).

As COVID-19 is likely to remain a prevalent infectious disease, it is essential that we elucidate the association between this SARS-CoV-2 infections and neuroinflammatory conditions such as NMOSD. Through this systematic review, we will assess the association between SARS-CoV-2 infections and the para and post-infectious manifestations of NMOSD. We will also investigate the potential association between COVID-19 vaccination and the development of *de novo* or relapsing NMOSD.

## Methods

### Design

Literature was retrieved from the following databases on September 13, 2022: Medline (Ovid), Cochrane Library (WileyOnline), Embase (Elsevier), Trip Database Pro, Clinicaltrials.gov, and Scopus (Elsevier). This systematic review was carried out in accordance with Preferred Reporting Items for Systematic Reviews and Meta-analysis (PRISMA) guidelines. We aimed to identify relevant articles reporting on NMOSD manifestations following a SARS*-*CoV*-*2 infection or a *de novo* or relapsing forms of NMOSD presenting in association with any type of approved COVID-19 vaccine.

### Search strategy

The search strategy combined keywords and controlled vocabulary related to NMOSD and COVID-19 and was tailored to the specifications of each database (see Supplementary appendix 1). A detailed search strategy can be found in Supplementary material. A manual search of bibliographies of relevant studies was also conducted. All citations for this review were required to be indexed in the peer-reviewed literature. Results were carefully verified to avoid duplicates or overlapping publications.

### Inclusion criteria

We identified and triaged manuscripts and included all peer-reviewed, full-text, English language manuscripts that reported cases of NMOSD that met the 2015 International Panel for NMOSD Diagnosis (IPND) criteria in association with SARS-CoV-2 infection or a COVID-19 vaccination (13).

### Exclusion criteria

The review was restricted to studies published in English. Poster and symposium abstracts, non-peer reviewed publications, and clinical trials were excluded from this report. We also excluded review papers, editorial, hypothesis reports, and commentaries, unless there was a report of a case of NMOSD following a SARS-CoV-2 infection or COVID-19 vaccine. Studies were also excluded if they contained insufficient clinical data, if the data was repeated from an article that had already been included, or if they addressed peripheral nervous system (PNS) demyelinating diseases or CNS demyelinating disorders other than NMOSD such as myelin oligodendrocyte glycoprotein antibody disease (MOGAD), TM, ON, MS, and acute disseminated encephalomyelitis (ADEM). Cases involving other types of coronaviruses (e.g., SARS-CoV/MERS-CoV) infections were also excluded.

### Data extraction

Titles and abstracts of all identified studies were independently screened for relevance by two reviewers, MW and TH, to ensure they met criteria for inclusion. Following a full-text screening of eligible articles, articles meeting criteria were retrieved, summarized, and managed on Covidence^®^ software. Discordant abstract or article decisions and screening queries were resolved by consensus. The same reviewers then extracted data on the following parameters: article title, authors, publication year, country, age/gender of the patients, aquaporin-4 (AQP4) antibody status, SARS-CoV-2 infection presentation, NMOSD clinical presentation, COVID-19 vaccine related information, interval prior to onset of neurological symptoms, MRI findings, cerebrospinal fluid (CSF) analysis, SARS-COV-2 laboratory findings, treatment, and clinical outcome.

### Statistical analysis

Quantitative data were described using range (minimum and maximum), mean and median, while qualitative data were described in percentages and numbers. Covidence software was used for evaluating and adjudicating articles for the systematic review and Microsoft Excel was used for statistical assessments.

## Results

**Figure 1:**
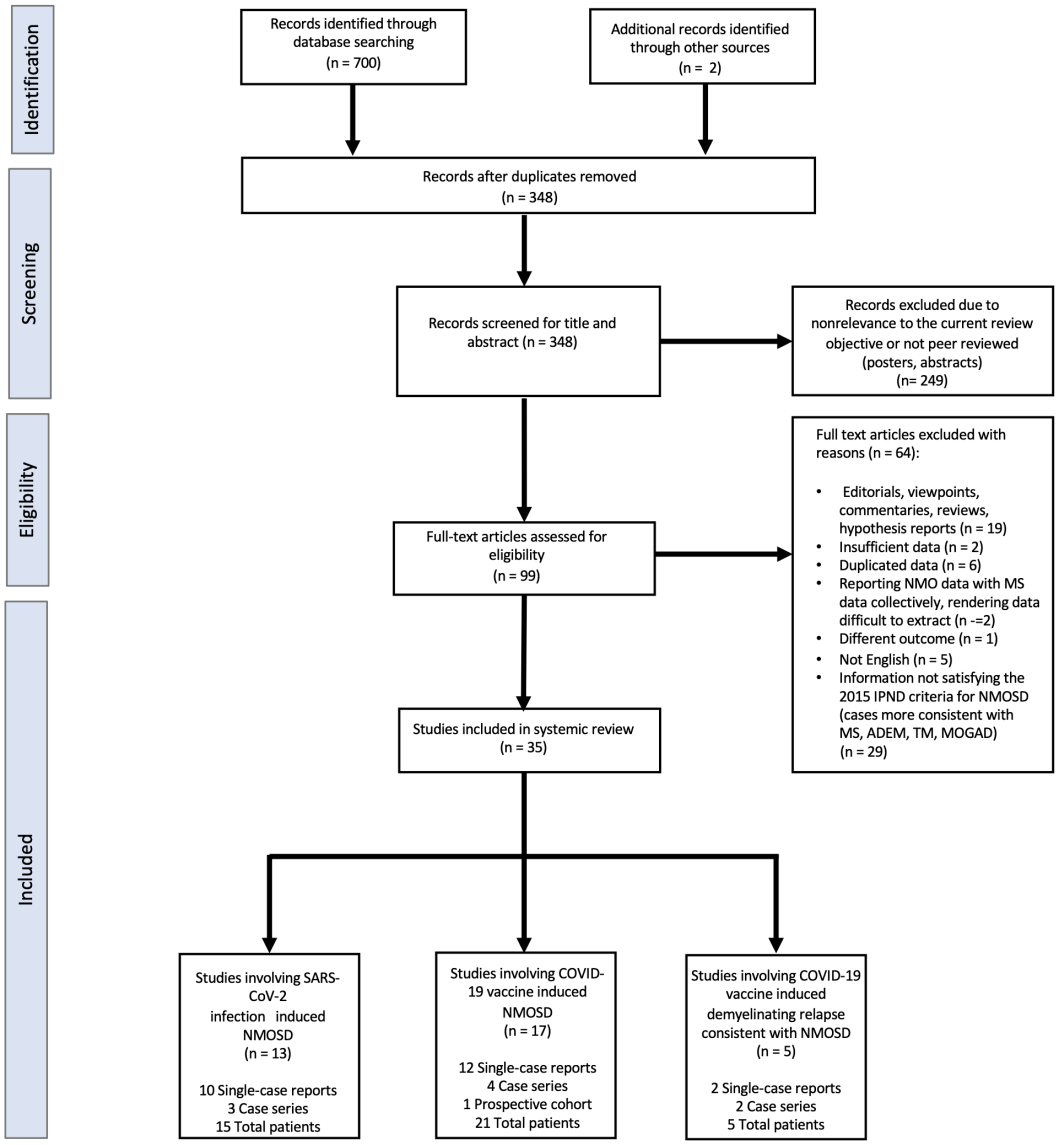
Flow chart of literature inclusion in accordance with PRISMA guidelines. ADEM, Acute Demyelinating Encephalomyelitis; IPND, International Panel for NMO Diagnosis; MOGAD, Myelin oligodendrocyte glycoprotein antibody-associated disease; MS, Multiple Sclerosis; NMOSD, Neuromyelitis Optica Spectrum Disorder; SARS-CoV-2, Severe Acute Respiratory Syndrome Coronavirus-2; TM, Transverse Myelitis.

As seen in the PRISMA flowchart (Figure 1), our systematic search identified 702 potentially relevant articles through various databases. A total of 354 duplicate articles were discarded. The remaining 348 articles were screened by title and abstract, and 249 non peer reviewed or nonrelevant articles were removed. Thereafter, a total of 34 studies were deemed eligible by the authors after applying the inclusion/exclusion criteria to the full text documents, of which there were 24 single-case reports, 9 case-series, and 1 prospective cohort study. These 34 reports described 41 unique patients which were divided into three categories: NMOSD onset following a SARS-CoV-2 infection, NMOSD onset following COVID-19 vaccination, and relapses consistent with NMOSD following COVID-19 vaccination. The clinical characteristics for each of these categories are presented in Tables 1–4 which summarizing the demographic and clinical characteristics of patients with SARS-CoV-2 post-infection and COVID-19 post-vaccination NMOSD manifestations.

**Table 1:** Characteristics of cases presenting with Neuromyelitis Optica in relation to a SARS-CoV-2 infection.

Reference/country	Age/sex	Comorbidities	Clinical presentation of COVID-19 infection	Clinical presentation of NMO	AQP4 antibody status^*^	Laboratory investigations	Time interval between COVID-19 and NMO (days)	MRI data	CSF findings	COVID-19 related findings	Treatment of NMO	Outcome
1. Aubart et al. (14) France	14/F	Juvenile arthritis	Asymptomatic	Monocular optic neuritis	+ (AQP4 Ab test not specified)	NR	NR	*Optic Nerves:* Optic Neuritis *Brain:* Spared Spine: Spared	NR	Positive SARS-CoV-2 nasal PCR	IVMP	Improvement
2. Barone et al. (15) Italy	35/M	None	NR	Monocular optic neuritis Myalgias	+ (AQP4 Ab test not specified)	ANA 1:640 Anti-TPO > 1,300 U/mL	30	*Optic Nerves:* Enhancing left optic nerve and optic chiasm lesion *Brain:* Spared Spine: Spared	NR	Negative Positive SARS-CoV-2 nasal PCR Positive serological IgG/IgM	IVIM IVIG Rituximab	Complete recovery
3. Batum et al. (16) Turkey	50/F	None	Fever Cough	Numbness, Urinary retention Weakness	+ (CSF AQP4-IgG)	Anti-CMV IgM negative, Brucella agglutination negative, EBV IgM negative, Anti-HAV IgM negative, Anti-HBc IgM negative, HIV negative, RF negative, ANA negative, ANCA negative, anti-mitochondrial antibody negative, Anti-smooth muscle antibody negative, Anti-Ro negative, Anti-La negative, Anti-ds DNA negative, Anti-nRNP negative, anti-Histon antibody negative, anti-MOG negative	NR	*Brain:* Spared Spine: LETM from C3 to Conus	Pleocytosis Protein 159 mg/dL OCB negative IgG index 1.2	Negative SARS-CoV-2 nasal PCR CXR: Bilateral Consolidation with ground-glass density	IVMP IVIG PLEX	Improvement
4. Correa et al. (7) Brazil	51/F	NR	Fever Myalgia Headache Anosmia Ageusia Cough	Myalgia Numbness Dysesthesias Weakness	+ (Serum and CSF cell-based assay for AQP4 antibodies positive)	ANA 1:320, Meningitis/Encephalitis Panel negative	14	*Brain:* Anterior fornix and subfornical organ lesions *Spine:* Enhancing LETM	Pleocytosis Elevated Protein Positive IgG index	Positive serological IgM	IVMP PLEX Azathioprine	Improvement
5. Das et al. (17) India	16/F	None	NR	Monocular optic neuritis Back and lower extremity stiffness	+ (Serum AQP4 IgG)	Vitamin B12 normal, thyroid hormone assay normal, serum anti-MOG negative, ANA positive, anti-Ro positive	~120	*Optic Nerves: Optic nerve lesion* *Brain: Fr*ontal subcortical area lesion Spine: LETM from C2 to C7	Normal WBC Normal Protein Elevated IgG index	Positive serological IgG/IgM	IVMP Rituximab	Improvement
6. Ghosh et al. (5) India	20/M	None	Fever Nausea/emesis Cough	Weakness Numbness Urinary retention Constipation Hiccups Nausea Vomiting Myalgias	+ (Transfected HEK293 cell-based assay serum AQP4 IgG positive)	CSF and paired sera: HIV, bacterial and parasitic infections, tuberculosis, autoimmune encephalitis and paraneoplastic encephalitis negative Serum studies: Systemic lupus erythematosus, Sjogren syndrome, Bechet’s disease, sarcoidosis, and antiphospholipid antibody syndrome negative, Anti-MOG antibodies	5	*Brain:* Spared *Spine:* Non enhancing LETM from the medulla to T12	WBC 10 cells/uL Protein 80 mg/dL Negative OCB	Positive SARS-CoV-2 nasal PCR	IVMP Rituximab	Improvement
7. Jentzer et al. (18) France	71/F	Hereditary Hemorrhagic Telangiectasia	NR	Paraplegia	+ (Serum anti-AQP4 semi-quantitative cell- based assay)	NR	~90	*Spine:* LETM from C7 to T6	NR	Positive SARS-CoV-2 nasal PCR	NR	NR
8. Khair et al. (6) United States	13/F	Suspected ADEM ADHD	Fatigue Anosmia Ageusia	Diffuse weakness	+ (Serum and CSF)	SS-B IgG antibody positive, Anti-MOG negative, MBP, viral PCR panel and autoimmune encephalopathy panel negative	~60	*Brain:* Numerous non-enhancing lesions in the brain and brainstem *Spine: Numerous* non-enhancing lesions in the cervical and thoracic spinal cord	NR	positive SARS-CoV-2 nasal PCR	IVMP	Improvement
9. Kim et al. (19) Korea	37/F	None	None	Bilateral lower extremity paraparesis Paresthesia Diminished deep tendon reflexes	+ (Serum AQP4 IgG)	Serum studies: C-reactive protein 7.09, Erythrocyte sedimentation rate 74 mm/h, VDRL negative, HIV negative, vitamins B1, B6, B12 normal, methylmalonic acid normal, thyroid-stimulating hormone normal, T3 normal, hemoglobin A1c normal, Jo-1 normal, SS-A/Ro negative, SS-B/La negative, double-stranded DNA negative, paraneoplastic antibodies negative, anti-ganglioside antibodies negative, immunofixation negative CSF Studies: CMV negative, *Mycobacterium tuberculosis* negative, *Mycoplasma pneumoniae* negative, varicella-zoster virus negative, herpes simplex virus type I and II negative, *Streptococcus pneumoniae* negtaive, *Neisseria meningitidis* negative, Hemophilus influenzae type 1 negative, *Listeria monocytogenes* negative, Group B streptococcus negative, and Cryptococcus negative	3	*Spine:* Enhancing LETM from C1/2 to conus medullaris	WBC 602 cell/uL Proteins 188.4 mg/dL IgG index 0.98 Oligoclonal bands negative Myeline basic protein negative	Positive SARS-CoV-2 nasal PCR	IVMP	Improvement
10. Mirmosayyeb et al. (20) UAE	43/F	None	Fatigue/asthenia Myalgias Anorexia	Urinary retention, Lower extremity numbness Thoracic sensory level Quadriplegia, Bilateral optic neuritis	− (AQP4 Ab test not specified)	NR	NR	*Optic Nerves:* Enhancing bilateral optic nerves *Brain:* Lesions in the thalami, brainstem, periaqueductal grey. Temporal lobe tumefactive lesion *Spine:* Enhancing LETM lesions throughout the cervical and thoracic cord	Mild pleocytosis Highly elevated myelin-basic protein Negative OCB	Positive SARS-CoV-2 PCR IgM/IgG	IVMP PLEX	Improvement
11. Mirmosayyeb et al. (20) United States	NR	NR	NR	Area postrema syndrome	+ (AQP4 Ab test not specified)	NR	NR	*Brain:* Dorsal medullary lesion *Spine:* LETM extending greater than 3 segments	NR	Positive SARS-CoV-2 nasal PCR Positive serological IgG/IgM	NR	NR
12. Mirmosayyeb et al. (20) Egypt	56/F	Surgically resected temporal meningioma	Fatigue Myalgias Anorexia Cough	Bilateral optic neuritis, Disorientation	NR (AQP4 Ab test not specified)	NR	14	*Brain:* Diencephalic, Thalami, Optic Chiasm, Optic Tracts lesions *Spine:* Spared	NR	Positive SARS-CoV-2 nasal PCR CXR: Bilateral Patchy Ground-Glass Opacification	IVMP	Died
13. Rafique et al. (21) Pakistan	7.5/F	None	None	Optic neuritis, Ataxia, Hypotonia, Hyporeflexia	− (Serum AQP4 IgG)	Anti-MOG antibody negative, anti-ganglioside antibody panel negative. CRP elevated, serum ferritin 497 ng/mL, LDH 376 U/L, ESR normal, D- Dimers 0.34 μg/mL	11	*Optic Nerves:* Optic nerve lesion *Brain:* Brain stem, area postrema, periaqueductal lesions *Spine:* Enhancing LETM cervical and thoracic lesions	NR	Positive serological IgG	IVMP IVIG PLEX	Improvement
14. Sardar et al. (22) Qatar	38/F	Diabetes Obesity Obstructive sleep apnea, Migraine Gastritis	Headache Nausea/emesis	Bilateral optic neuritis, Holocephalic headache, Nausea	− (AQP4 Ab test not specified)	NR	14	*Optic nerves:* Bilateral optic nerve lesions *Brain:* Spared *Spine:* Spared	Normal WBC Normal Protein Oligoclonal bands negative	Positive SARS-CoV-2 nasal PCR	IVMP PLEX	Improvement
15. Shaw et al. (23) Australia	70/M	Hypertension Heart disease GERD Former smoker	Dyspnea	Visual blurring, Ptosis, Weakness, Urinary Incontinence, Fasciculation	− (AQP4 Ab test not specified)	C-reactive protein 282 mg/L	9	*Brain:* Spared *Spine:* Enhancing patchy multifocal T5 to T11 lesions	NR	Positive SARS-CoV-2 nasal PCR Positive serological IgG CXR: Bilateral patchy ground-glass opacification	None	Intubated/died

Ab, antibody; ADEM, acute disseminated encephalomyelitis; ADHD, attention-deficit/hyperactivity disorder; ANA, anti-nuclear antibody; ANCA, anti-neutrophile cytoplasmic antibodies; AQP4, aquaporin-4; CMV, cytomegalovirus; CSF, cerebral spinal fluid; C, cervical; CXR, chest x-ray; EBV, epstein-Bar virus; F, female; HAV, hepatitis A virus; HBV, hepatitis B virus; HIV, human immunodeficiency virus; IgM, immunoglobulin M; IVIG, intravenous immunoglobulins; IVMP, intravenous methylprednisolone; LETM, longitudinally extensive transverse myelitis; M, male; NMO, neuromyelitis optica; PCR, polymerase chain reaction; MRI, magnetic resonance imaging; NR, not reported; PLEX, plasmapheresis; RF, rheumatoid factor; T, thoracic; VDRL, venereal disease research laboratory; WBC, white blood cells.

*This column documents whether the AQP4 antibody testing was completed in the serum, CSF or both and whether it was completed as a cell based assay or an Elisa study. If not specified in this column, the original manuscript did not list this information.

**Table 2:** Characteristics of cases presenting with *de novo* neuromyelitis optica in relation to COVID-19 vaccination.

Reference/country	Age/sex	Comorbidities	Name of vaccine (Vaccine type)	Dose #	Time interval between vaccination and NMO (days)	Clinical presentation of NMO	MRI data	CSF findings	AQP4 antibody status*	Laboratory investigation	Treatment of NMO	Outcome
1. Anamnart et al. (24) Thailand	26/F	None	Sinovac CoronaVac (Inactivated COVID-19 vaccine)	1	10	Leg monopoiesis, Decreased pinprick sensation in the arm, trunk, and leg, Generalized hyperreflexia	*Brain:* Spared *Spine:* Enhancing C4 to C5 lesion	Normal WBC Normal Protein Oligoclonal bands negative	+ (Serum AQP4- IgG by cell-based indirect immunofluorescence assay (CBA-IIF, Euroimmun^®^), titer 1:320)	NR	IVMP PLEX Rituximab	Improvement
2. Anamnart et al. (24) Thailand	46/F	None	Oxford–AstraZeneca ChAdOx1 nCoV-19 (Viral vector vaccine)	1	9	Unilateral lower extremity weakness and hypesthesia, Hyperreflexia	*Brain:* Non-enhancing Medulla and subependymal periventricular area lesions *Spine: Enhancing* C2 to C3 lesion	Normal WBC Normal Protein Oligoclonal bands negative	+ (Serum AQP4- IgG by cell-based indirect immunofluorescence assay (CBA-IIF, Euroimmun^®^), titer 1:320)	NR	IVMP Azathioprine	Improvement
3. Arora et al. (25) India	50/M	None	NR (Vital vector vaccine)	1	20	Bilateral upper and lower extremity weakness, Urinary retention, Bilateral vision loss	*Brain:* Non-enhancing bilateral dorsolateral thalamic lesions *Spine:* C1, C2, T8 lesions	WBC 32 cells/uL Protein 55 mg/dL Oligoclonal bands negative	+ (Serum AQP4-IgG)	ANA negative, C-ANCA negative, P-ANCA negative, VDRL, negative. ACE levels normal. Anti- MOG antibodies negative	IVMP IVIG	Improvement
4. Badrawi et al. (26) United Arab Emirates	34/M	None	Sputnik V COVID-19 (Adenovirus viral vector vaccine)	2	21	Acute confusions, Dizziness, Headache, Imbalance	*Optic nerves:* Optic chiasm lesion *Brain:* Extensive periventricular and/or peri-ependymal lesions including along the third and fourth ventricles and periaqueductal gray mater. Lesions in the thalamus and corpus callosum *`Spine:* Spared	Lymphocystis Elevated protein. Oligoclonal bands negative HSV negative, Syphilis negative, cryptococcal antigen negative, VZV negative	+ (Serum AQP4-IgG Titer 1:40)	COVID-19 negative, adenovirus negative, Herpes Simplex virus (type I & II) negtaive, Epstein Barr virus negative, Cytomegalovirus, and Human Immunodeficiency virus negative	PLEX	Improvement
5. Ballout et al. (27) United States	63/F	Hyperthyroid Hyperlipidemia	*Pfizer*-*BioNTech* COVID-19 *Vaccine* (*BNT162b2*) (mRNA vaccine)	1	7	Weakness Urinary retention	*Brain: Enhancing* Thalamic lesion *Spine:* Non-enhancing central LETM from T6 to T12	WBC 33 cells/uL Protein 57 mg/dL	+ (Serum AQP-4 IgG Utilizing ELISA technique and CSF anti AQP4 Ab CBA with a titer of 1:16)	ANA 1:2560, Anti-DsDNA IU/mL, AE normal, C3 and C4 complement normal, paraneoplastic panel negative, CSF anti-MOG ab negative	IVMP PLEX	Improvement
6. Ballout et al. (27) United States	54/F	Immune thrombocytopenia purpura	Moderna SARS-CoV-2 mRNA-1,273 vaccine	2	3	Ascending numbness	*Brain:* Spared *Spine:* Enhancing central LETM from T2 to T9l	WBC 26 cells/u: Protein 71 mg/dL MBP 27 Oligoclonal bands negative	+ (Serum AQP-4 IgG Utilizing ELISA technique with titers of 1,417.3 U/mL and CSF anti AQP4 Ab CBA)	ANA 1:320, ESR normal, CRP normal, c-ANCA normal, p-ANCA normal, ACE normal, SSA negative, SSB negative, serum and CSF anti-MOG negative, DsDNA antibodies negative	IVMP	Improvement
7. Caliskan et al. (28) Turkey	43/F	None	*Pfizer*-*BioNTech* COVID-19 *Vaccine* (*BNT162b2*) (mRNA vaccine)	NR	1	Monocular optic, neuritis Hemiparesthesia, Hemiparesis, Urinary retention, Constipation	*Optic nerve:* Unliteral optic neuritis *Brain:* Enhancing periatrium lesion Non enhancing left crus cerebri *Spine:* Patchy enhancing lesion from C1 to mid-thoracic level	WBC 6 cels/uL Protein 40.1 mg/dL Oligoclonal bands positive	+ (Serum AQP-4 IgG Utilizing CBA with a titer of 1:320)	ANA negative, DsDNA antibody negative, lupus anticoagulant negative, RF negative, anti-cardiolipin antibody, and anti-beta2 glycoprotein levels normal, HIV negative, CMV negative, hepatitis viruses negative, VZV negative, CA 12–5 normal, CA 19–9 normal, CA 15–3, normal, human epididymis protein 4 normal, Anti-MOG ab negative	IVMP PLEX	Complete recovery
8. Chen et al. (29) China	Middle aged/F	None	Probable Sinovac CoronaVac or Sinopharm vaccine (Inactivated COVID-19 vaccine)	1	3	Emesis, Dizziness, Unsteady gait	*Brain:* Non enhancing area postrema and bilateral hypothalamus lesions *Spine:* Spared	WBC 31 cell/uL Normal Protein Oligoclonal bands negative	+ (Serum AQP-4 IgG Utilizing CBA)	Vitamin B1 & B12 levels normal, tumor markers normal, ESR normal, CRP normal, immunoglobulins normal, complements normal, RF negative, antiphospholipid antibodies negative, GFAP IgG negative, Autoimmune encephalitis antibodies negative, paraneoplastic antibodies negative, serum cytokines (IFN-γ, IL-6, IL-4, IL-2, IL-10, IL-21, TNF-ɑ) normal, ANA, positive SSA positive, SSB positive, Ro-52 positive, and p-ANCA positive	IVMP	Improvement
9. Fujikawa et al. (30) United States	46/F	Vitamin B12 deficiency	Moderna SARS-CoV-2 mRNA-1,273 vaccine (mRNA vaccine)	1	2	Shooting back pian, Paresthesia distal to the T10 dermatome, Bilateral upper and lower extremity weakness Urinary retention	*Brain:* Spared *Spine:* Non-enhancing LETM from C6-T2	Normal WBC Normal Protein Oligoclonal bands negative	− (AQP-4 IgG test not specified)	Vitamin B12 level 245 pg./m, CRP normal, TSH normal, hemoglobin A1C normal, aldolase normal, methylmalonic acid normal, antinuclear antibody normal, Jo-1 normal, SS-A/Ro negative, SS-B/La negative, ribonucleoprotein normal, scleroderma negative, DsDNA negative, anti-ribosomal, chromatin normal, centromere B antibodies negative, C3 & C4 compliments normal	IVMP	Improvement
10. Janarious et al. (31) United States	19/F	None	Moderna SARS-CoV-2 mRNA-1,273 vaccine (mRNA vaccine)	NR	15	Bilateral upper and lower extremity weakness and sensory changes, Urinary incontinence, T4 sensory level	*Brain:* NR *Spine:* LETM from Cervicomedullary junction to the conus medullaris	Pleocytosis Increased IgG synthesis rate	+ (CSF AQP-4 IgG positive, Serum AQP-4 Ab negative)	Serum Anti-MOG Ab negative	IVMP PLEX Rituximab	NR
11. Kim et al. (32) Korea	47/F	None	Oxford–AstraZeneca ChAdOx1 nCoV-19 (Viral vector vaccine)	1	22	Intractable hiccups, Gait disturbance, Dysarthria, Dysphagia, Hoarseness	*Brain:* Enhancing medullary lesion, Non-enhancing parietal periventricular lesion *Spine:* Spared	WBC 0 cells/uL Protein 27 mg/dL Oligoclonal bands negative IgG index 0.44	+ (Serum AQP-4 IgG)	NR	IVMP Azathioprine	Complete recovery
12. Kim et al. (32) Korea	57/F	Sjogren’s syndrome	Moderna SARS-CoV-2 mRNA-1,273 vaccine (mRNA vaccine)	1	11	Constipation, Bilateral lower extremity paresthesia, T-12 hypoesthesia sensory level, Unilateral diminished position sensation, Bilateral lower extremity diminished vibration sensation, Spasticity	*Brain:* Non-specific white matter changes *Spine:* Enhancing LETM from T5–T9	WBC 0 cells/uL Protein 31 mg/dL Oligoclonal bands negative	+ (Serum AQP-4 IgG)	NR	IVMP Azathioprine	Improvement
13 Kim et al. (19) Korea	37/F	None	*Pfizer*-*BioNTech* COVID-19 *Vaccine* (*BNT162b2*) (mRNA vaccine)	3	19	Bilateral lower extremity paraparesis, Paresthesia, Diminished deep tendon reflexes	*Brain:* Spared *Spine:* Enhancing intramedullary LETM from C1 to the conus medullaris	WBC 602 cells/uL Proteins 188.4 mg/dL IgG index 0.98 Oligoclonal bands negative	+ (AQP-4 IgG test not specified)	CRP 7.09, ESR 74 mm/h, VDRL negative, HIV negative, vitamins B1, B6, & B12 normal, methylmalonic acid normal, thyroid-stimulating hormone normal, T3 normal, hemoglobin A1c normal, Jo-1 negative, SS-A/Ro negative, SS-B/La negative, DsDNA negative, paraneoplastic antibodies negative, anti-ganglioside antibodies negative	IVMP	Improvement
14. Khayat-Khoei et al. (33) Germany	64/M	Sjogren’s disease	*Pfizer*-*BioNTech* COVID-19 *Vaccine* (*BNT162b2*) (mRNA vaccine)	1	18	Pain, Paresthesia, Unilateral weakness, Urinary retention, Constipation, Balance/gait impairment, Saddle anesthesia	*Brain:* Non-enhancing corpus callosum, frontal white mater, parietal white mater lesions *Spine:* Enhancing central LETM from cervical spine to conus	WBC 1 cells/uL Protein 39 mg/dL Oligoclonal bands negative IgG index 0.68	+ (serum AGP-4 IgG titer > 1:100,000, CSF AQP-4 IgG titer1:128)	Positive SS-A/SS-B antibodies	IVMP PLEX	Improvement
15. Kuntz et al. (34) Canada	80/M	NR	*Pfizer*-*BioNTech* COVID-19 *Vaccine* (*BNT162b2*) (mRNA vaccine)	2	2	Unilateral weakness, Unilateral numbness, Gait instability Urinary retention	*Brain:* Spared *Spine:* LETM from T3-T4 to T9-T10	WBC 39 cells/uL Protein Normal Oligoclonal bands negative	+ (serum AGP-4 IgG positive)	Anti-MOG Ab positive on initial test and negative on repetition, CRP 10.9, Serological screening for rheumatological and infectious diseases was unremarkable	IVMP PLEX, Mycophenolate mofetil	Improvement
16. Lévi-Strauss et al. (35) France	72/F	None	Moderna SARS-CoV-2 mRNA-1,273 vaccine (mRNA vaccine)	1	7	Paresthesia, Hypoesthesia Weakness of the left arm and leg, Alteration of consciousness, Left sided choreoathetosis	*Brain:* Non-enhancing corpus callosum, area postrema, and periependymal lesions *Spine:* Spared	WBC 500 cells/uL Protein 117 mg/dL Oligoclonal bands negative	+ (serum AGP-4 IgG positive via CBA)	HIV negative, No no immunodeficiency profile completed, ANA 1:160, anti-SSA/Ro antibody titer > 8 UI/mL, anti-DNA negative, anti-phospholipid antibodies negative, ANCA negative. Anti-MOG negative, anti-thyroid antibodies negative, CSF antiparaneoplastic panel (NMDA, anti-AMPA and anti-VGKC) negative, Serum antiparaneoplastic panel (anti-Yo, -Ri, -GAD, -Hu, -CV and -Tr antibodies) negative	IVMP PLEX Rituximab	Improvement
17. Motahharynia et al. (36) Iran	70/F	None	Sinovac CoronaVac (Inactivated COVID-19 vaccine)	3	7	Unilateral upper and lower extremity hypoesthesia, Quadriplegia	*Brain:* Spared *Spine: Enhancing rim shaped enhancing hemorrhagic LETM from C1 to C7. Lesion from T1 to T3*	WBC normal Protein normal Oligoclonal bands negative	+ (Serum AQP4- IgG via CBA)	NR	IVMP PLEX, Cyclo-phosphamide	Death
18. Shirah et al. (37) Saudi Arabia	31/F	Systemic Lupus Erythematosus	*Pfizer*-*BioNTech* COVID-19 *Vaccine* (*BNT162b2*) (mRNA vaccine)	NR	14	Monocular optic neuritis	*Optic nerve:* Enhancing intraocular and intraorbital segments of the left optic nerve *Brain:* Spared *Spine:* Spared	WBC normal Protein normal Oligoclonal bands negative	+ (Serum AQP-4 IgG via immunofluorescence test with a titer of 1:1000)	ANA positive, DsDNA positive (968 IU/mL), ANCA positive, Anti-SSA positive (109 EU/mL) Anti-SSB positive (128 EU/mL), Low C3 (0.72 g/L) & C4 (0.08 g/L) compliments	IVMP PLEX, Rituximab	No recovery
19. Tasci et al. (38) Turkey	32/M	Graves’ Disease Gastric neuroendocrine tumor	Sinovac CoronaVac (Inactivated COVID-19 vaccine)	1	14	Unilateral optic neuritis	*Optic nerves:* Right optic Neuritis *Brain;* Spared *Spine:* Spared	NR	+ (Serum AQP-4 IgG)	NR	IVMP Rituximab	Improvement
20. Tisavipat et al. (39) Thailand	50/M	None	Oxford–AstraZeneca ChAdOx1 nCoV-19 (Viral vector vaccine)	2	4	Quadriparesis, Painful tonic spasms, Urinary retention	*Brain;* Spared *Spine:* Enhancing LETM from C2 to T1	NR	+ (Serum AQP-4 IgG)	NR	IVMP Rituximab	Improvement
21. Tisavipat et al. (39) Thailand	70/F	None	Oxford–AstraZeneca ChAdOx1 nCoV-19 (Viral vector vaccine)	1	10	Lhermitte’s sign, Unliteral arm weakness	*Brain;* Spared *Spine:* LETM from C1 to T1	NR	+ (Serum AQP-4 IgG)	NR	IVMP	Improvement

Ab, antibody; ACE, angiotensin converting enzyme; ANA, antinuclear antibodies; ANCA, anti-neutrophil cytoplasm antibodies; AQP4, aquaporin-4; ANCA, antineutrophil cytoplasmic antibodies; CBA, cell based assay; CMV, cytomegalovirus; CRP, C-reactive protein, CSF, cerebral spinal fluid; C, cervical; CXR, chest x-ray; DsDNA, double-stranded deoxyribonucleic acid; ELISA, Enzyme-linked immunosorbent assay; ESR, erythrocyte sedimentation rate; F, female; HSV, herpes Simplex virus; HIV, human immunodeficiency virus; IVIG, intravenous immunoglobulins; IVMP, intravenous methylprednisolone; LETM, longitudinally extensive transverse myelitis; M, male; MOG, myelin oligodendrocyte glycoprotein; MRI, magnetic resonance imaging; NMO, neuromyelitis optica; NR, not reported; PCR, polymerase chain reaction; PLEX, plasmapheresis; RF, rheumatoid factor; SSA, anti-sjogren's syndrome A; SSB, anti-sjogren's syndrome B; T, thoracic; TSH, thyroid-stimulating hormone; VDRL, venereal disease research laboratory; VZV, varicella zoster virus; WBC, white blood cells.

*This column documents whether the AQP4 antibody testing was completed in the serum, CSF or both and whether it was completed as a cell-based assay or an Elisa study. If not specified in this column, the original manuscript did not list this information.

**Table 3:** Characteristics of cases presenting with central nervous system relapses consistent with neuromyelitis optica in relation to COVID-19 vaccination.

Reference/country	Age/sex	Pre-existing history of CNS autoimmune disease	Name of vaccine (vaccine type)	Dose #	Time interval between vaccination & NMO (days)	Clinical presentation of NMO	MRI data	CSF findings	AQP4 antibody status*	Laboratory investigations	Treatment of NMO	Outcome
1. Dinoto et al. (40) Italy	38/F	AQP4 + NMO on rituximab	*Pfizer-BioNTech* *COVID-19 Vaccine (BNT162b2)* (mRNA vaccine)	2	10	Optic neuritis	NR	NR	+ (AQP4 Ab test not specified)	NR	IVMP	Complete recovery
2. Dinoto et al. (40) Italy	61/F	AQP4 + NMO not on a DMT	*Pfizer*-*BioNTech* COVID-19 *Vaccine* (*BNT162b2*) (mRNA vaccine)	2	97	Myelitis	NR	NR	+ (AQP4 Ab test not specified)	NR	IVMP	No recovery
3. Fragoso et al. (41) Brazil	62/F	NMOSD DMT status not reported	Oxford–AstraZeneca ChAdOx1 nCoV-19 (Viral vector vaccine)	1	7	Monocular vision loss	*Optic* nerve: Enhancing unliteral optic nerve lesion *Brain:* Spared *Spine:* Spared	NR	NR	NR	IVMP PLEX	Improvement
4. Helmchen et al. (42) Germany	40/F	Multiple sclerosis on natalizumab	Oxford–AstraZeneca ChAdOx1 nCoV-19 (Viral vector vaccine)	1	14	Binocular blindness, Lower extremity numbness, T5 sensory level, Back pain, Incontinence, Paraplegia	*Optic nerve:* Enhancing lesion in the chiasm and bilateral optic nerves and tracts *Brain:* Spared *Spine:* LETM from C7 - T1, LETM from T7 - T10, medullary conus lesion	WBC 524 cells/uL Protein 220 mg/dL	-	Anti-MOG negative (confirmed via indirect immuno-fluorescence testing with MOG-transfected HEK-293 cells), GFAP negative, flotillin negative, ANA negative, anti-phospholipids ab negative	IVMP	Improvement
5. Lohmann et al. (43) Germany	68/F	Secondary progressive multiple sclerosis Not on a DMT	*Pfizer-BioNTech* *COVID-19 Vaccine (BNT162b2)* (mRNA vaccine)	1	23	Sensorimotor paraparesis with a T8 level, Bowel and bladder incontinence	*Brain:* NR *Spine:* Enhancing LETM from C4 to T10	WBC 340 cells/uL Protein 259 mg/dL Oligoclonal bands negative	+ (CSF and serum AQP-4 IgG)		IVMP PLEX Eculizumab	Improvement

Ab, antibody; ANA, anti-nuclear antibody; AQP4, aquaporin-4; CSF, cerebral spinal fluid; C, cervical; CXR, chest x-ray; DMT, disease modifying therapy; F, female; GFAP, glial fibrillary acid protein; IVIG, intravenous immunoglobulins; IVMP, intravenous methylprednisolone; LETM, longitudinally extensive transverse myelitis; M, male; MOG, myelin oligodendrocyte glycoprotein; MRI, magnetic resonance imaging; NMO, neuromyelitis optica; NR, not reported; PCR, PLEX, plasmapheresis; T, thoracic; WBC, white blood cells.

*This column documents whether the AQP4 antibody testing was completed in the serum, CSF or both and whether it was completed as a cell based assay or an Elisa study. If not specified in this column, the original manuscript did not list this information.

**Table 4:** Comparison of demographic and disease characteristics of patients with SARS-CoV-2 post-infection and COVID-19 post-vaccination NMOSD.

Characteristics	NMOSD following a SARS-CoV-2 infection	*De novo* and relapsing NMOSD following COVID-19 vaccination
Age in years, mean (SD)	37.5 (21)	50 (16)
**Sex**		
Female (%)	11 (73%)	20 (77%)
Male (%)	3 (20%)	6 (33%)
Not reported	1 (7%)	0 (0%)
Patients with a reported comorbid autoimmune condition (%)	2 (13%)	8 (31%)
Patients with a comorbid condition	6 (40%)	12 (46%)
Days between exposure to SARS-CoV-2 infection vs. COVID-19 vaccination & NMOSD onset (range)	14 (3–120)	10 (1–97)
**Neurological manifestations**		
Transverse myelitis	10 (67%)	17 (65%)
Short-segment transverse myelitis	2 (13%)	4 (15%)
Longitudinally extensive transverse myelitis	8 (53%)	13 (50%)
Optic neuritis	7 (47%)	5 (19%)
Area postrema syndrome	2 (13%)	3 (12%)
Brainstem involvement	5 (33%)	3 (12%)
**AQP-4 antibody status**		
Positive (%)	10 (67%)	22 (85%)
Negative (%)	4 (27%)	3 (12%)
Unknown (%)	1 (7%)	1 (4%)
**Outcome**		
Complete or partial recovery	11 (73%)	22 (85%)
No recovery	0 (0%)	2 (8%)
Death (%)	2 (13%)	1 (4%)
Not reported	2 (13%)	1 (4%)

### New onset NMOSD following SARS-CoV-2 infection

Of the 15 patients that developed NMOSD following SARS-CoV-2 infection, 11 were female (73%), 3 were male (20%), and one was not identified (7%). The reported cases came from 12 countries; 2 cases each from France, India, and the United States of America (USA), and 1 case each from Italy, Korea, Pakistan, Qatar, Turkey, the United Arab Emirates, Australia, Brazil, and Egypt. Given a total of 626,337,158 world-wide COVID-19 cases as of October 31, 2022 (2), the global incidence based on reported cases of NMOSD following a SARS-CoV-2 infection is 0.02 per million.

The median age of the patients was 37.5 years (range 7.5–71 years). The latency period from the onset of COVID-19 symptoms to the first neurological manifestations followed a dual distribution: (i) *Short latency*: 3 to 14 days in 8/15 patients (53%) and (ii) *Long latency* (60 to 120 days) in 3/15 patients (20%). The median time interval between the initial SARS-CoV-2 infection symptoms and NMOSD symptom onset was 14 days (range 3–120 days).

Interestingly, 2/15 (13%) of patients had a history of a previously diagnosed immune-mediated condition; one patient had juvenile arthritis, and the other patient had a past episode of suspected ADEM. Comorbidities were present in 6/15 patients (40%) and are summarized with other clinical characteristics in Table 1.

In terms of the clinical presentation, TM was the most common neurological phenotype occurring in 10/15 (67%) patients. Two of the 10 had short-segment TM (STM) spanning over less than 3 vertebral segments and 8 were longitudinally extensive TM (LETM) spanning 3 or more vertebral segments. The second most common presentation was ON, found in 7 (47%) patients. APS, defined as intractable nausea, vomiting, or hiccups persisting for at least 48 h, was found in 2 (13%) patients. Brainstem involvement was found in 5 (33%). Ten patients (67%) tested positive for AQP4 antibody, while 4 (27%) were AQP4 antibody negative (one case not reported). CSF analysis in this group demonstrated pleocytosis in 5/15 (33%) patients while 2/15 (13%) had normal white blood cell (WBC) counts. CSF findings were not reported for 8/15 (53%) patients. High protein levels were reported in 2/15 (13%) patients.

Of the 13 cases that reported on acute treatment, all but one patient (92%) was initially treated with intravenous methylprednisolone. In additional to methylprednisolone, 5/13 (39%) were treated with plasmapheresis and 3/13 (23%) were treated with intravenous immunoglobulin (IVIG). Maintenance immunotherapy was provided to only four patients, including rituximab (*n* = 3) and azathioprine (*n* = 1). The treatment outcomes were reported for 13 of the 15 patients. Of these patients, 11/13 (84%) experienced complete or partial recovery following treatment, while 2/13 (15%) patients died. One death was caused by multiorgan failure and sepsis secondary to the SARS-CoV-2 infection. The second patient died from respiratory insufficiency, lymphopenia, and fever following cyclophosphamide treatment.

### New onset and relapsing NMOSD following COVID-19 vaccination

Tables 2, 3 describe the clinical presentation, laboratory and imaging findings, and treatment outcomes of both *de novo* and relapsing NMOSD cases following the COVID-19 vaccine.

After receiving a COVID-19 vaccination, 26 patients developed a new demyelinating event related to NMOSD. A total of 21 of the 26 (81%) cases experienced an initial relapse of NMOSD following the COVID-19 vaccination, while 5 of the 26 (19%) cases had a recurrent exacerbation attributed to NMOSD following vaccination. Of the 5 relapsing cases, 3 of the patients had a known diagnosis of NMOSD, while two patients had been initially diagnosed with MS, which was unmasked as NMOSD post vaccination. Of note, one of the patients with known NMOSD had been stable and relapse free for 8 years, prior to their vaccine inducing a new relapse.

Based on data from the WHO, a total of 12,830,378,906 vaccine doses have been administered globally as of October 31, 2022 (2), the global incidence of an NMOSD demyelinating events among reported cases in the literature following vaccination is 0.002 per million.

Of the 26 cases developing NMOSD manifestations following a COVID-19 vaccination, 9 cases (35%) occurred after receiving the Pfizer-BioNTech BNT162b2 mRNA vaccine, 6 (23%) following the Oxford–AstraZeneca ChAdOx1 nCoV-19 viral vector vaccine, 5 (19%) following the Moderna mRNA-1273 vaccine, 4 (15%) after the Sinovac or Sinopharm inactivated COVID-19 vaccine, and 1 (4%) following the Sputnik V adenovirus viral vector vaccine. The specific vaccine involved in one case was unspecified, but it was a viral vector vaccine. In sum, 54% (14/26) of cases involved an mRNA vaccine, 31% (8/26) of cases involved a viral vector vaccine, and 15% (4/26) of cases involved an inactivated COVID-19 vaccine.

In terms of demographics, there was a female preponderance with a 3.3:1 ratio of female to male cases. The median age was 50 years with an age range of 19 to 80. The reported cases came from 13 countries with 4 cases each from Thailand and the USA, 3 cases each from Germany and Korea, 2 cases each from Italy and Turkey, and 1 case each from Brazil, Canada, China, France, India, Iran, Saudi Arabia, and the United Arab Emirates.

**Figure 2:**
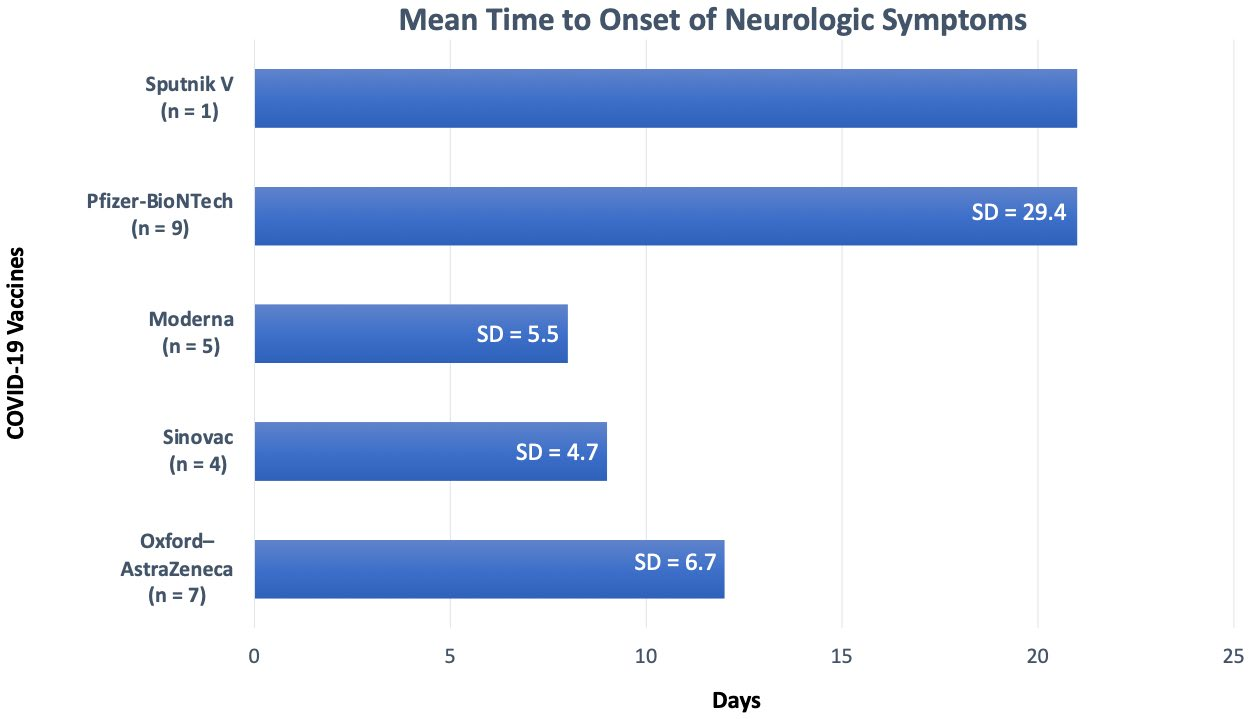
Duration from vaccination to symptom onset for each vaccine. SD, Standard deviation.

The median duration between vaccination and onset of NMOSD related clinical symptoms was 10 days (range 1–97 days). Figure 2, displays time intervals between vaccination and neurological symptom onset for each COVID-19 vaccine. Breaking down symptom onset with dose of the vaccine, 15/26 (58%) patients, experienced the onset of neurological symptoms following the first dose of the vaccine. A total of 6/26 (23%) patients had the onset of neurological symptoms following the second dose of the vaccine, and in 2/26 (8%) patients, the onset of neurological symptoms followed the third dose of the vaccine. One case did not specify, which dose induced the neurological symptoms.

Interestingly, 8/26 (31%) patients had a history of a previously diagnosed immune-mediated condition. In addition, 4/26 (15%) patients reported a family history of an immune-mediated condition including MS, AQP4-IgG positive NMOSD, myasthenia gravis, and systemic lupus erythematous.

Turning to the clinical presentation, TM was the most common phenotype, occurring in 17 (65%) patients. Four of those were STM spanning over less than 3 vertebral segments and 13 were LETM spanning 3 or more vertebral segments. The second most common presentation was ON, found in 5 (19%) patients. APS was found in 3 (12%) patients and brainstem involvement was found in 3 (12%) patients.

Of the 25 patients with a reported AQP4 antibody status, 22/25 (88%) patients tested positive for AQP4 antibody, while 3 (12%) were AQP4 antibody negative. Of the 20 patients with reported CSF results 11/20 (55%) had pleocytosis, 9/20 (45%) had elevated CSF protein levels. Only one patient’s CSF findings were positive for oligoclonal bands (OCB) out of the 16 cases that explicitly documented OCB status.

In terms of therapy, all but one patient (96%) was initially treated with intravenous methylprednisolone. Subsequently, 12/26 (46%) patients were treated with pulses of plasmapheresis, and 1/26 (4%) patients was treated with IVIG. Maintenance immunotherapy was documented in 12/26 (46%) patients, including rituximab (*n* = 6), azathioprine (*n* = 3), cyclophosphamide (*n* = 1), eculizumab (*n* = 1), and mycophenolate mofetil (*n* = 1). The treatment outcomes were reported for 25/26 patients. Of these patients, 22/25 (88%) experienced complete or partial recovery following treatment, 2/25 (8%) patients did not improve with treatment, and 1/25 (4%) patients died. The cause of death was not discussed in the case series.

### Comparison of demographic and clinical characteristics of patients with SARS-CoV-2 post-infection and COVID-19 post-vaccination NMOSD

Table 4 compares the demographic and clinical characteristics of NMOSD following SAR-CoV-2 infection and COVID-19 vaccination. The COVID-19 vaccine exposure group and the SARS-CoV-2 viral infection group had similar sex ratios with a female preponderance, but the vaccine group’s age was on average over a decade older than the SARS-CoV-2 infected group. Both groups had a similar percentage of comorbidities, but the COVID-19 vaccine group (31% vs. 13%) was more likely to present with a comorbid autoimmune condition. Both groups had a similar rate of transverse myelitis, but the SARS-CoV-2 infected group were more likely to present with optic neuritis and brain stem involvement. The COVID-19 vaccinated group was also more likely to present with positive AQP4 antibodies than the SARS-CoV-2 infected group (85% vs. 65%). Both groups demonstrated a similar mortality rate.

## Discussion

As the COVID-19 pandemic has continued to persist, a mounting number of neurological manifestations and complications related to this disease have been described. Para and post infectious and post vaccination autoimmune CNS demyelination is a rare, but well documented phenomena. A small but accumulating base of literature suggests an association between the SARS-CoV-2 infection, the COVID-19 vaccine, and NMOSD. This systemic review contributes to this growing literature, including 41 worldwide cases of NMOSD temporally associated with the SARS-CoV-2 infection or COVID-19 vaccination. The analysis revealed that the NMOSD cases met standardized criteria, neurological symptoms developed within 2 weeks in most cases, the majority responded to standard immune therapies and overall neurological morbidity was moderate with 7% mortality.

The theory that a viral infection can trigger NMOSD pathogenesis is supported by several case series and case reports demonstrating an association between NMOSD and various viral infections including epstein barr virus, influenza virus, human immunodeficiency viruses (HIV), and varicella zoster virus (9, 44–47). SARS-CoV-2 infection has joined these other viral agents as a potential risk factor for PNS and CNS demyelinating disease (48, 49). In fact, TM, acute necrotizing encephalopathy, acute inflammatory demyelinating polyneuropathy (AIDP), and ADEM events have been associated with SARS-CoV-2 para and post infections, demonstrating that this emergent viral disease is associated with other CNS demyelinating disorders (50–53). Additionally, case reports have demonstrated an association between COVID-19 vaccinations and the onset of ADEM, TM, and MS following the COVID-19 vaccination (33, 54, 55).

The pathological mechanism explaining how the COVID-19 vaccine or the SARS-COV-2 infection induce NMOSD is not fully understood, but it is hypothesized that there is an interplay between viral and vaccine-related features and individual susceptibility factors (56). SARS-CoV-2 is thought to infect its host via the angiotensin-converting enzyme-2 (ACE-2) receptors on the cell surface of type II alveolar epithelial cells in the lung (57, 58). ACE-2 receptors are also expressed on the glial cells and the neurons (59). Therefore, in addition to infecting the respiratory system, SARS-CoV-2 can impact the central and peripheral nervous system. Once the host is exposed to either the COVID-19 vaccine or SARS-CoV-2 infection, NMOSD development may be mediated by either neurotropism or via aberrant immune mediated injury (5). Once SARS-CoV-2 has accessed the nervous system, several proposed pathological mechanisms have been suggested including bystander activation, spreading of the epitope, molecular/immunological mimicry involving cross-reactive autoantibodies targeting SARS-CoV-2 antigens, and amplified blood–brain barrier (BBB) permeability allowing antibody (i.e., AQP-4 peptides) entry into the CNS (5, 10). Evidence indicates that SARS-CoV-2 crosses the blood brain barrier (BBB) along with other cytokines including IL-1β, IL-2, IL-4, IL-6, IL-8, IL-10, TNF-α, and IFN-γ. This impacts macrophages, microglia, and astrocytes, which mediate a cytokine storm leading to the death of neurons and oligodendrocytes. This produces a cytokine storm and a proinflammatory state. Of these cytokines, IL-6 has particular significance as it has been implicated in playing a critical role in regulating the immune response in MS by promoting pathogenic T helper (Th) 17 cells generation (60). Disruption of Th 17 and regulatory T cell responses caused by SARs-CoV-2 exposure can induce inflammation and mitochondrial dysfunction that amplifies the inflammatory process, resulting in immune-metabolic constraints on neural energy metabolism (61). Additional mechanisms include activation of toll-like receptors (TLRs), antibody production against myelin via molecular mimicry, and the affinity for angiotensin-converting enzyme 2 (ACE2) receptors, which can induce myelin destruction (62). Furthermore, neuro-invasion by SAR-CoV-2 or its antigens may cause leakage of CNS antigens such as AQP-4 peptides into the systemic circulation, triggering the bystander immune cascade (5).

Several case reports have indicated that cytotoxic lesion of the corpus callosum (CLOCCs) are also associated with COVID-19 vaccinations (63–65). CLOCCs is caused by an influx of water into the cells due to cytokine induced glutamate release from astrocytes (63). The proposed underlying pathogenic mechanism between COVID-19 vaccine triggered NMOSD and CLOCCS is similar. For example, the CSF of CLOCCS patients is notable for elevated IL-6 and IL-10 and these cytokines are also implicated in NMOSD induction. Similarly, Toll-like receptors, which are activated by mRNA vaccines, have been implicated in both processes and both disorders respond to intravenous IV IgG and corticosteroids. Cytokine storm pathology is a central mechanism of both vaccine induced NMOSD and CLOCCs (65).

In terms of SARS-CoV-2 variants in our case series, it is difficult to assess which types were most often implicated. None of the individual case reports discussed which variant was responsible for the reported COVID-19 case associated with NMOSD onset. Except in one case, the original case reports and case series, did not document the date of infection, rendering it difficult to assess which variant was the dominant strain at the time. Furthermore, the publications that reviewed cases of SARS-Cov-2 associated NMOSD were published in 10 different countries across a 3 three-year time span. Using either the date of publication or the date the paper was received to determine the latest possible date that each case of SARS-CoV-2 infection, we found 8 cases reported in 2020, 3 cases in 2021, and 4 case in 2022. Given the diversity of locations and the range of dates of publication and the failure of these publications to document the date of infection, it is not possible to provide reliable data on which variants were represented in this case series. That said, most cases would have contracted the earlier pre-Omicron variants of SARS-CoV-2 (66).

The latency period between vaccine or infection exposure and NMOSD clinical onset ranged from 1 to 120 days but the majority of patients developed neurological symptoms within 1–2 weeks following exposure to the virus or the vaccine. In order for a disorder to be considered vaccine induced, the WHO suggests that there should be a clear temporal relationship between exposure and disease onset. The latency period between the exposure and the adverse event, however, was not defined by the WHO (67). Other studies that attempted to demonstrate a causal link between vaccination and disease onset included various latency time ranges from 8 weeks to 5 months (68, 69). For example, Karussis et al. (68) completed a PubMed search from 1979 to 2013 reviewing 71 documented cases of post-vaccination CNS demyelination secondary to various vaccines including influenza, HPV, and hepatitis A or B vaccines. In their review, symptoms typically manifested within 2 weeks (mean: 14.2 days), however, they also included delayed presentations from 4 weeks to 5 months post-vaccination (68). One study assessing the association between hepatitis B vaccination and the development of MS between 1991 and 1997 utilized an 8-week latency period between vaccination exposure and disease onset (69). Given the rarity of NMOSD, in our study, we included a delayed latency period of up to 120 days to ensure completeness of the data. However, the majority of the cases presented with a latency period of less than 30 days. The mean latency period between SARS-CoV-2 infection and NMOSD development was 34 days [Standard deviation (SD) 39 days]. Of the 11 cases that reported the latency period, only 3 were over 30 days. Of the 21 patients that developed *de novo* NMOSD following the COVID-19 vaccine, all patients had a latency period of less than 30 days (mean: 10 days). Of the patients that developed a relapsing CNS demyelination consistent with NMO following exposure to the COVID-19 vaccine, only one of the 5 cases presented with a latency period of more than 30 days (mean: 30 days). This short-term association, however, should be considered with reservations as there are no controls or quantitative risk outcomes (e.g., odds ratios).

The cases presenting with a long latency distribution, in which NMOSD occurred more than 28 days after the exposure, may represent coincidental NMOSD manifestations. In the long latency cases, the vaccine or infectious exposure and NMOSD disease onset may be causally related rather than causative. These cases of prolonged latency may represent sporadic NMOSD that may have occurred regardless of the exposure, especially as both the SARS-CoV-2 infections and COVID-19 vaccinations were wide spread over a brief interval and a large portion of the population encountered at least one of these exposures. The cases with a short latency distribution of less than 28 days are less likely to be coincidental, although causation cannot be proven. Both short and long latency periods were included, however, for completeness as this is a hypothesis generating study. We advise a case-controlled study for a more rigorous investigation.

The current data, spanning from December 2019 to the present provides too brief of an overview to give insight into the long-term risks of para-post infectious and post vaccine associated NMOSD. The data suggests, however, that if SARS-CoV-2 or COVID-19 vaccine exposed patients meet the diagnostic criteria for NMOSD, they should be managed like any other NMOSD patient to optimize the clinical outcome.

Females comprised the majority (76%) of cases in this series. This female preponderance corresponds with data in the literature that indicates a 2-fold higher incidence among females with NMOSD compared with males (70). The female preponderance found in our series may be secondary to a heightened immune response against self and foreign antigens in females compared to males.

With 24% of cases having a prior immune-mediated condition, *de novo* and relapsing NMOSD manifestations may be more prevalent among those with a pre-existing autoimmune disease. The results of this review suggest that in some susceptible individuals, exposure to the SARS-CoV-2 infection or COVID-19 vaccine may introduce a short-term risk of CNS demyelination.

Although this review indicates that there is a plausible association between the COVID-19 vaccination and NMOSD, the number of cases appears to be rare, and vaccination is still strongly encouraged. Currently, epidemiological and clinical data suggests that the benefits of vaccination conferred to both the individual and the public supersedes the possible risk of NMOSD associated with vaccination (34, 71). Furthermore, given the large number of patients that have received the COVID-19 vaccination, only a few reports have documented NMOSD manifestations following the vaccine, indicating that this is an uncommon occurrence.

This is a comprehensive systemic review of NMOSD cases associated with SARS-CoV-2 infections and the COVID-19 vaccine, including 34 published reports and 41 individual cases. The majority of cases reported in the existing literature were presented as case reports, and the few case series available were often more broadly focused on a variety of CNS demyelinating disorders rather than exclusively discussing NMOSD.

Given the established temporal relationship between SARS-CoV-2 infections and COVID-19 vaccination and the onset of NMOSD, our systemic review adds the current literature that underscores a potential link between viral infections and vaccinations and the development of *de novo* and relapsing NMOSD. This review suggests a probable association between post-infectious or vaccine triggered autoimmune mediated CNS demyelinating astrocytopathy. Our findings also suggest that vaccine and viral triggered CNS autoimmune demyelination may be more common among individuals with a pre-existing autoimmune disorder or a family history of autoimmune disease. However, the heterogeneity of the clinical data prevents a metanalysis from being performed. Although a causative relationship cannot be established on a temporal association alone, raising awareness of this potential correlation may influence the diagnosis and management of future patients presenting with demyelinating sequalae in the setting of infectious or vaccine mediated triggers. The lack of a control group prevented our ability to generate standard risk outcomes and future studies involving a control group are merited. This paper provides evidence for hypothesis generation that can be further tested with a case-control study allowing for a more detailed characterization of demographic, clinical characteristic, and genetic data to prove causality.

Strengths of this review include the comprehensive search of the literature, the detailed adjudication of cases and the comparison of COVID-19 vaccine and SARS-CoV-2 infection. Limitations included the small number of cases with retrospective observations. Several cases included incomplete workups and there was heterogeneity of clinical data available, impairing the ability to complete a meta-analysis.

## Conclusion

This systematic review comprehensively demonstrates a temporal association between *de novo* and relapsing forms of NMOSD and SARS-CoV-2 infections and COVID-19 vaccinations. Association, however, does not away imply causation. We would also emphasize that the protective benefits that the COVID-19 vaccine conveys to both the individual and society as a whole far outweigh any hypothetical risk that would be implied from this review. Our report suggests, a link between the COVID-19 virus or vaccine exposure and the pathological cascade that may induce clinical NMOSD symptoms. Furthermore, given the brief duration of the study, the potential long-term effects of exposure are unknown. This systematic review does suggest that NMO manifestations following a COVID-19 viral or vaccine exposure may be more common than currently recognized, particularly among high-risk demographic groups. This association requires further study using quantitative epidemiological assessments in representative populations to better quantify the risk of developing clinical symptoms of NMOSD.

## Author contributions

MW and TH contributed to conceptualization, study design, literature search, obtaining data, data management and analyses, data verification, drafting the manuscript and figures, manuscript revisions, statistical analysis, administrative oversight, study supervision, and validation. All authors contributed to the article and approved the submitted version.

## Conflict of interest

The authors declare that the research was conducted in the absence of any commercial or financial relationships that could be construed as a potential conflict of interest.

## Publisher’s note

All claims expressed in this article are solely those of the authors and do not necessarily represent those of their affiliated organizations, or those of the publisher, the editors and the reviewers. Any product that may be evaluated in this article, or claim that may be made by its manufacturer, is not guaranteed or endorsed by the publisher.
