# Supplementary material for: New onset or relapsing neuromyelitis optica temporally associated with SARS-CoV-2 infection and COVID-19 vaccination: a systematic review
Source: Front Neurol. 2023 Jun 22;14:1099758. doi: 10.3389/fneur.2023.1099758 (PMC10323143; doi:10.3389/fneur.2023.1099758)
Supplement: Supplementary file 1 [file Data_Sheet_1.docx]

**Appendix 1 – Database Search Strategies**

Search strategies developed by Emily F. Gorman, MLIS and reviewed by Emilie Ludeman, MSLIS

Searches run on 13 September 2022

**MEDLINE and Epub Ahead of Print, In-Process, In-Data-Reviews & Other Non-Indexed Citations and Daily (Ovid, 1946 – September 9, 2022) – 122** references retrieved on 13 September 2022

*Multi-line search run in the Advanced Search interface:*

1. exp neuromyelitis optica/
2. (neuromyelitis optica or nmo or nmosd or myelooptic neuropathy or devic* disease or devic* syndrome or myeloopticoneuropathy or myelopticoneuropathy or myeloptico neuropathy or neuropticomyelitis or optic neuromyelitis).ti,ab,kw.
3. 1 or 2
4. exp covid-19/ or exp sars-cov-2/ or coronavirus infections/
5. (coronavirus* or corona virus* or coronavirinae or coronaviridae or betacoronavirus or hcov or 2019ncov or cov 2 or cov2 or covid or covid19 or covid2019 or ncov or novel cov or sars2 or sarscov2 or ((wuhan or hubei) adj3 (virus or respiratory or pneumonia or outbreak* or epidemic or pandemic))).ti,ab,kw.
6. 4 or 5
7. 3 and 6

**Embase (Embase.com) – 306** references retrieved on 13 September 2022

*One-line search run in the Results tab of the Embase.com interface:*

('myelooptic neuropathy'/exp OR 'neuromyelitis optica':ti,ab,kw OR nmo:ti,ab,kw OR nmosd:ti,ab,kw OR 'myelooptic neuropathy':ti,ab,kw OR 'devic* disease':ti,ab,kw OR 'devic* syndrome':ti,ab,kw OR myeloopticoneuropathy:ti,ab,kw OR myelopticoneuropathy:ti,ab,kw OR 'myeloptico neuropathy':ti,ab,kw OR neuropticomyelitis:ti,ab,kw OR 'optic neuromyelitis':ti,ab,kw) AND ('coronavirus disease 2019'/exp OR 'coronavirus infection'/de OR coronavirus*:ti,ab,kw OR 'corona virus*':ti,ab,kw OR coronavirinae:ti,ab,kw OR coronaviridae:ti,ab,kw OR betacoronavirus:ti,ab,kw OR hcov:ti,ab,kw OR 2019ncov:ti,ab,kw OR 'cov 2':ti,ab,kw OR cov2:ti,ab,kw OR covid:ti,ab,kw OR covid19:ti,ab,kw OR covid2019:ti,ab,kw OR ncov:ti,ab,kw OR 'novel cov':ti,ab,kw OR sars2:ti,ab,kw OR sarscov2:ti,ab,kw OR (((wuhan OR hubei) NEAR/3 (virus OR respiratory OR pneumonia OR outbreak* OR epidemic OR pandemic)):ti,ab,kw))

**Cochrane Library (WileyOnline; Cochrane Database of Systematic Reviews, Cochrane Central Register of Controlled Trials, Cochrane Methodology Register) – 2** references retrieved on 13 September 2022 [0 reviews, 2 trials]

*Using Search Manager in Advanced Search*

1. [mh "neuromyelitis optica"]
2. ("neuromyelitis optica" or nmo or nmosd or "myelooptic neuropathy" or (devic* next disease) or (devic* next syndrome) or myeloopticoneuropathy or myelopticoneuropathy or "myeloptico neuropathy" or neuropticomyelitis or "optic neuromyelitis"):ti,ab,kw
3. #1 or #2
4. [mh "covid-19"] or [mh "sars-cov-2"] or [mh ^"coronavirus infections"]
5. (coronavirus* or (corona next virus*) or coronavirinae or coronaviridae or betacoronavirus or hcov or 2019ncov or "cov 2" or cov2 or covid or covid19 or covid2019 or ncov or "novel cov" or sars2 or sarscov2 or ((wuhan or hubei) near/3 (virus or respiratory or pneumonia or outbreak* or epidemic or pandemic))):ti,ab,kw
6. #4 or #5
7. #3 and #6

**Trip Database Pro – 38** references retrieved on 13 September 2022

("neuromyelitis optica" OR nmo OR nmosd OR "myelooptic neuropathy" OR "devic* disease" OR "devic* syndrome" OR myeloopticoneuropathy OR myelopticoneuropathy OR "myeloptico neuropathy" OR neuropticomyelitis OR "optic neuromyelitis") AND (coronavirus* OR "corona virus*" OR coronavirinae OR coronaviridae OR betacoronavirus OR hcov OR 2019ncov OR "cov 2" OR cov2 OR covid OR covid19 OR covid2019 OR ncov OR "novel cov" OR sars2 OR sarscov2)

**ClinicalTrials.gov – 1** reference retrieved on 13 September 2022

*Entered into “Condition or disease” box:* "neuromyelitis optica" OR nmo OR nmosd OR "myelooptic neuropathy" OR "devic* disease" OR "devic* syndrome" OR myeloopticoneuropathy OR myelopticoneuropathy OR "myeloptico neuropathy" OR neuropticomyelitis OR "optic neuromyelitis"

*Combined with the following in the “Other terms” box:* coronavirus* OR "corona virus*" OR coronavirinae OR coronaviridae OR betacoronavirus OR hcov OR 2019ncov OR "cov 2" OR cov2 OR covid OR covid19 OR covid2019 OR ncov OR "novel cov" OR sars2 OR sarscov2

**Scopus (Scopus.com) – 231** references retrieved on 13 September 2022

*One-line search run in the Advanced Search interface:*

TITLE-ABS-KEY("neuromyelitis optica" OR nmo OR nmosd OR "myelooptic neuropathy" OR "devic* disease" OR "devic* syndrome" OR myeloopticoneuropathy OR myelopticoneuropathy OR "myeloptico neuropathy" OR neuropticomyelitis OR "optic neuromyelitis") AND TITLE-ABS-KEY(coronavirus* or "corona virus*" or coronavirinae or coronaviridae or betacoronavirus or hcov or 2019ncov or "cov 2" or cov2 or covid or covid19 or covid2019 or ncov or "novel cov" or sars2 or sarscov2 or ((wuhan or hubei) W/3 (virus or respiratory or pneumonia or outbreak* or epidemic or pandemic)))
